# A Systematic Review and Meta-Analysis of the Proportion Estimates of Disseminated Intravascular Coagulation (DIC) in Malaria

**DOI:** 10.3390/tropicalmed8060289

**Published:** 2023-05-23

**Authors:** Thitinat Duangchan, Manas Kotepui, Suriyan Sukati, Yanisa Rattanapan, Kinley Wangdi

**Affiliations:** 1 Medical Technology, School of Allied Health Sciences, Walailak University, Tha Sala, Nakhon Si Thammarat 80160, Thailand; 2 Hematology and Transfusion Science Research Center, Walailak University, Tha Sala, Nakhon Si Thammarat 80160, Thailand; 3 Department of Global Health, National Centre for Epidemiology and Population Health, College of Health and Medicine, Australian National University, Canberra 2601, Australia

**Keywords:** malaria, DIC, disseminated intravascular coagulation, *Plasmodium*, systematic review, meta-analysis

## Abstract

Disseminated intravascular coagulation (DIC) is a potentially life-threatening condition that causes systemic coagulation to be turned on and coagulation factors to be used up. However, the evidence for DIC in malaria patients is still not clear, and small case series and retrospective studies have shown varying results. This meta-analysis was intended for the evaluation of the evidence of DIC among malaria patients using a meta-analysis approach. The protocol for the systematic review was registered at PROSPERO as CRD42023392194. Studies that investigated DIC in patients with malaria were searched in Ovid, Scopus, Embase, PubMed, and MEDLINE. The pooled proportion with 95% confidence intervals (CI) of DIC among malaria patients was estimated using a random-effects model. A total of 1837 articles were identified, and 38 articles were included in the meta-analysis. The overall proportion of DIC in malaria was 11.6% (95% CI: 8.9%–14.3%, I^2^: 93.2%, 38 studies). DIC in severe *falciparum* malaria and fatal malaria was 14.6% (95% CI: 5.0–24.3%, I^2^: 95.5%, 11 studies) and 82.2% (95% CI: 56.2–100%, I^2^: 87.3, 4 studies). The estimates of DIC among severe malaria patients who had multi-organ dysfunction with bleeding, cerebral malaria, acute renal failure, and ≥2 complications were 79.6% (95% CI: 67.1–88.2%, one study), 11.9% (95% CI: 7.9–17.6%, one study), 16.7% (95% CI: 10.2–23.3%, ten studies), and 4.8% (95% CI: 1.9–7.7%, nine studies), respectively. The proportion estimates of DIC among the patients with malaria depended on the *Plasmodium* species, clinical severity, and types of severe complications. The information from this study provided useful information to guide the management of malaria patients. Future studies are needed to investigate the association between *Plasmodium* infection and DIC and to understand the mechanism of malaria-induced DIC.

## 1. Introduction

Malaria is a parasitic infectious disease transmitted through the bites of female *Anopheles* mosquitoes infected with *Plasmodium* species. It is one of the deadliest infectious diseases in humans, which is frequently found in tropical and subtropical areas. Additionally, international travelers and immigrants from endemic countries are a known source of malaria and distribution worldwide [1,2]. Currently, five *Plasmodium* species have been reported to be responsible for malaria infection, being *Plasmodium falciparum*, *Plasmodium vivax*, *Plasmodium ovale*, *Plasmodium malariae*, and *Plasmodium knowlesi* [3]. Among these *Plasmodium* species, *P. falciparum* and *P. vivax* are the two main species that account for the most cases of malaria in humans. While *P. falciparum* causes severe malaria and is a major cause of mortality in malaria infection, *P. vivax* is the most widespread of all malaria species [4,5]. In 2021, it was estimated that more than 200 million people were infected with malaria, leading to 619,000 deaths [3]. Malaria infection causes the combination of any typical clinical symptoms of high fever, chills, headache, muscle aches, malaise, nausea, vomiting, and diarrhea [6]. Moreover, progressive malarial infection, usually caused by *P. falciparum*, can lead to serious manifestations such as severe and complicated malaria. Severe malaria is characterized by clinical and laboratory features that indicate vital organ dysfunction. It is defined as the presence of one or more of the following criteria: impaired consciousness, severe anemia, cerebral malaria, acute renal failure, pulmonary edema, hypoglycemia, jaundice, or bleeding. Complicated malaria, on the other hand, refers to severe malaria that is accompanied by other complications, such as shock, acute respiratory distress syndrome, and disseminated intravascular coagulation (DIC) [7,8,9].

Clinically apparent hemorrhage or DIC is a potentially life-threatening condition characterized by systemic and often uncontrolled coagulation activation and consumption of coagulation factors, resulting in blood clot formation throughout the circulation, organ dysfunction, clotting factor, and platelet depletion resulting in bleeding tendency [10]. The deposition of blood clots within the vessels then blocks and disrupts the blood flow to various organs, contributing to single or multiple-organ system dysfunction [11]. DIC is a result of a number of underlying conditions, including infection, sepsis, inflammation, trauma, cancer, and pregnancy [12]. The literature suggests that the complications of DIC could be associated with severe malaria and high mortality [8]. There is the consequence of mechanisms responsible for inducing DIC in malaria infection, including the exposure of the sub-endothelial matrix from capillary damage, the release of tissue factor into the blood circulation, the decrease in the synthesis of anticoagulants from the liver, and the induction of microparticles from activated platelets, red blood cells, as well as macrophages [13,14,15,16]. In addition, the hypercoagulable condition in malaria patients can lead to both microvascular thrombi and hemorrhagic manifestations [17]. There are some reports of the epidemiology of DIC in malaria infection [18,19]. However, the evidence for DIC in malaria patients is still not clear, and small case series and retrospective studies have shown conflicting results. Therefore, the goal of this systematic review and meta-analysis was to look at the evidence of DIC in patients with malaria. The information from this study may be useful for management, increase awareness for prevention, and guide treatment options for DIC among individuals with severe complications of malaria. 

## 2. Methods

### 2.1. Registration of a Systematic Review Protocol

The protocol of systematic review was registered at PROSPERO: CRD42023392194.

### 2.2. Guideline of Reporting Systematic Review

The reports of systematic review and meta-analysis followed the PRISMA statement (PRISMA Abstract Checklist, PRISMA 2020 Checklist) [20].

### 2.3. Research Question and Outcome

The systematic review followed the Condition, Context, Population (CoCoPop): Co, DIC; Co, worldwide; POP, patients with malaria [21].

### 2.4. Outcome

The outcome of the study was the pooled proportion estimate of DIC in patients with malaria.

### 2.5. Search Strategy

The search strategy was created by using search terms with the Boolean operators “AND” and “OR” as follows: “(“Disseminated Intravascular Coagulation” OR “Disseminated Intravascular” OR “Disseminated Coagulation” OR “Disseminated Coagulations” OR “Consumption Coagulopathy” OR “Consumption Coagulopathies” OR “Intravascular Coagulation” OR “Intravascular Coagulations”) AND (“Malaria” OR “*Plasmodium*” OR “Remittent Fever” OR “Marsh Fever” OR “Paludism”)”. For the searches in PubMed, the MeSH term was used as: “(((malaria) OR (malaria [MeSH Terms])) OR (*Plasmodium*)) OR (*Plasmodium* [MeSH Terms]) AND (((Disseminated Intravascular Coagulation) OR (Disseminated Intravascular Coagulation [MeSH Terms])) OR (Consumption Coagulopathy)) OR (Consumption Coagulopathy [MeSH Terms])”. The searches in other databases, including Ovid, Scopus, Embase, and MEDLINE, were slightly modified according to each database (Table S1). The results of the searches were imported into Endnote version 20.0 (Clarivate Analytics, Philadelphia, PA, USA).

### 2.6. Eligibility Criteria, Study Selection, Data Extraction

The original studies that investigated the DIC in patients with malaria from inception to 7 January 2023 were included in the present study. Studies without full texts, non-human studies such as animal studies, and in vitro studies were excluded. Non-original articles such as reviews, case reports, letters, notes, and systematic reviews were also excluded. The retrieved studies from the database searches were imported into Endnote version 20.0 (Clarivate Analytics, Philadelphia, PA, USA) for reference management. Before selecting the studies, duplicates were removed by both the automation tool in the Endnote software and manually by the authors. The remaining studies were screened for titles and abstracts and non-relevant studies were excluded. The eligible studies were then examined for full texts against the pre-set criteria. Study selection was performed independently by two authors (TD and MK). The disagreement over selection between authors was resolved by discussion. After the study selection was finalized, the necessary data were extracted into a Microsoft Excel sheet, including information on the name of the first author, publication year, study design, study area, participants (and number), clinical status (complications), age groups, *Plasmodium* spp., methods for *Plasmodium* detection, total number of patients with malaria, criteria for DIC, method for DIC, number of patients with DIC, and DIC score. Data extraction was performed independently by two authors (TD and MK). The disagreement over selection between authors was finalized by discussion for consensus.

### 2.7. Quality of the Included Studies

The studies were assessed for their reporting quality based on Strengthening the Reporting of Observational Studies in Epidemiology (STROBE) criteria for observational studies [22]. The STROBE criteria assessed the study’s quality in terms of the title, abstract, introduction, methods, results, and discussion sections of the articles with an overall 22 items. The study’s quality was judged as low, moderate, or high quality if the study passed <50%, 50–75%, or >75% of the total items, respectively. The quality of the included studies was determined independently by two authors (TD and SS). The disagreement over assessment between authors was finalized by another author (MK).

### 2.8. Meta-Analysis

The pooled proportion with 95% confidence intervals (CI) of DIC among malaria patients was estimated using a random-effects model as described previously by DerSimonian and Laird [23]. Forest plots were constructed to demonstrate the distribution of proportion point estimates and their 95% CI for the outcomes. Inconsistency index (I^2^-statistic) values of 0–40%, 30–60%, 50–90%, and 75–100% indicated unimportant, moderate, substantial, and considerable heterogeneity, respectively [24]. If the meta-regression showed any significant value for covariates such as publication years, study design, continent, age group, *Plasmodium* spp., method for malaria detection, or clinical status, sub-group analyses were performed. The funnel plot and Egger’s test were employed to detect possible publication bias. All analyses were performed using the statistical software Stata Version 17.0 (StataCorp., College Station, TX, USA).

## 3. Results

### 3.1. Search Results

**Figure 1:**
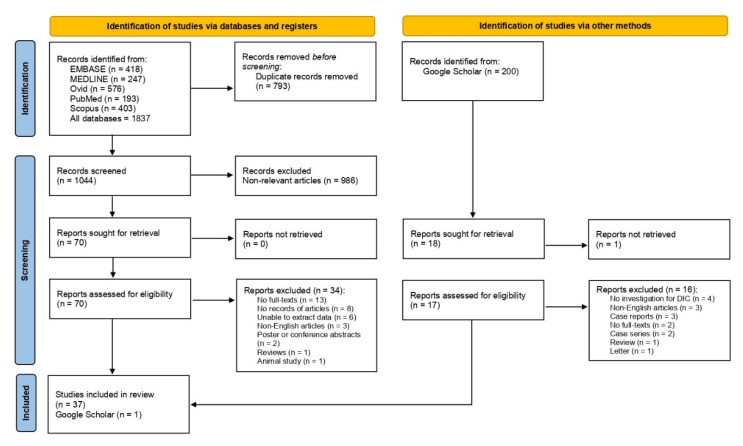
Study selection diagram. The figure shows the study selection processes, including the study selection from main databases and Google Scholar.

A total of 1837 articles were identified from Embase (n = 418), MEDLINE (n = 247), Ovid (n = 576), PubMed (n = 193), and Scopus (n = 403). After 793 duplicates had been removed, 1044 articles were screened for their titles and abstracts. Then, 986 non-relevant articles, such as articles that enrolled other diseases rather than malaria were excluded. The remaining 70 articles were further examined for eligibility, and 34 articles were excluded for the following reasons: no full-texts (n = 13), no records of articles (n = 8), unable to extract data (n = 6), non-English articles (n = 3), poster or conference abstracts (n = 2), reviews (n = 1), and an animal study (n = 1). A total of 37 studies [25,26,27,28,29,30,31,32,33,34,35,36,37,38,39,40,41,42,43,44,45,46,47,48,49,50,51,52,53,54,55,56,57,58,59,60,61] met the criteria and were included in the study. Another study [62] was identified from searches on Google Scholar. Finally, 38 studies [25,26,27,28,29,30,31,32,33,34,35,36,37,38,39,40,41,42,43,44,45,46,47,48,49,50,51,52,53,54,55,56,57,58,59,60,61,62] were included in the study for final analysis (Figure 1).

### 3.2. Summary Feature of Studies

A summary of the included studies is shown in Table 1. Most of the studies were cross-sectional (31.6%), prospective observational (26.3%), and retrospective observational (26.3%). Half of the studies were published between 2010 and 2022 (50%). Studies were conducted in six countries in Asia (71.1%) [25,26,28,29,31,32,33,35,36,37,38,39,40,41,43,45,46,48,49,50,51,54,58,59,60,61,62], four countries in Europe (10.5%) [34,42,52,56], two countries in Africa (7.89%) [27,44,47], North America (the United States) [30,55,57], and South America and Asia (Brazil and India) [53]. Most studies enrolled patients infected with *P. falciparum* (44.7%) and more than half of them in adult participants (60.5%). More than half of the studies enrolled patients with severe malaria (57.9%), followed by severe and non-severe malaria (34.2%). Microscopy was the most used method for the detection of *Plasmodium* (65.8%). Details of the studies are shown in Table S2.

### 3.3. Quality of the Included Studies

According to the STROBE criteria that assessed the study’s quality with an overall score of 22 items, two studies received high scores [47,53]. The remaining 22 studies had moderate scores [25,26,28,32,35,36,37,39,40,42,43,44,48,49,50,51,52,55,57,58,59,61], and 14 studies had low scores [27,29,30,31,33,34,38,41,45,46,54,56,60,62], respectively. All studies were included in the meta-analysis of the proportion. 

### 3.4. Proportion Estimates of DIC in Malaria

Six studies investigated the evidence of DIC in patients with malaria but no DIC was found [27,29,30,41,51,56]. The highest proportion of DIC in patients with malaria (79.6%) was reported in the study by Das et al. that investigated the occurrence of DIC in malaria patients with multi-organ dysfunctions and stratified the patients into overt bleeding and without overt bleeding [32]. The high proportion of DIC in patients with malaria was followed by Milner Jr et al. who investigated the evidence of DIC in patients with cerebral malaria or severe malarial anemia (proportion percentage = 62.5%) [44]. The authors stratified the patients into cerebral malaria with sequestration of parasitized red blood cells in the brain, cerebral malaria with sequestration of parasitized red blood cells in the brain and the presence of cerebral microthrombi, ring hemorrhages and extraerythrocytic malaria pigment, cerebral malaria with no sequestration of parasitized red blood cells in the brain, and patients with severe malarial anemia [44]. The remaining studies reported the proportion of DIC in patients with malaria being between 0.6% and 33.7% [25,26,28,31,33,34,35,36,37,38,39,40,42,43,45,46,47,48,49,50,52,53,54,55,57,58,59,60,61,62].

**Figure 2:**
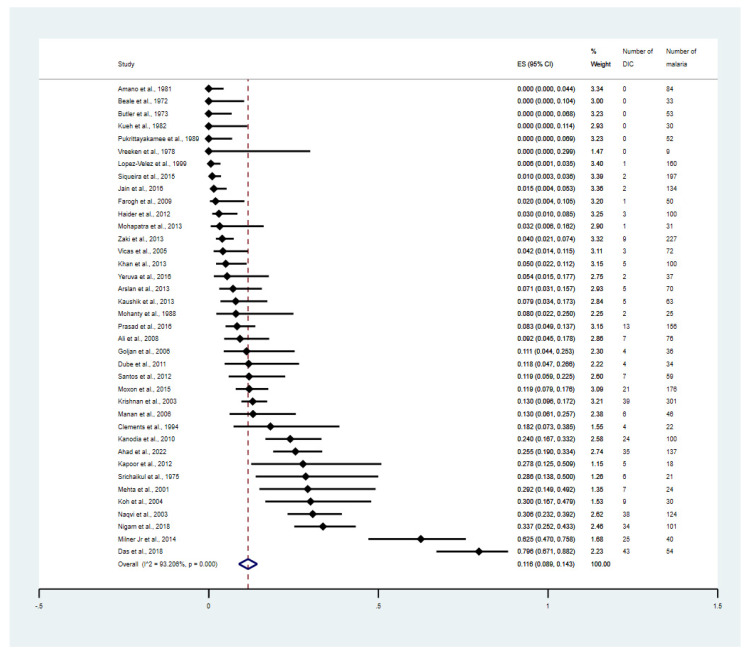
Proportion estimates of DIC among patients with malaria. The figure shows the proportion estimates of DIC in patients with malaria in an individual study (×100 unit) and also the pooled proportion estimates of DIC in patients with malaria. Abbreviations: DIC, disseminated intra-vascular coagulation; CI, confidence interval; ES, proportion estimates; I^2^, inconsistency index; *p*, significance value of Chi-square test for heterogeneity; % weight, contribution of individual study to the pooled proportion estimate [25,26,27,28,29,30,31,32,33,34,35,36,37,38,39,40,41,42,43,44,45,46,47,48,49,50,51,52,53,54,55,56,57,58,59,60,61,62].

The overall proportion of DIC in malaria was estimated using the data from 38 studies [25,26,27,28,29,30,31,32,33,34,35,36,37,38,39,40,41,42,43,44,45,46,47,48,49,50,51,52,53,54,55,56,57,58,59,60,61,62]. The results showed that the overall proportion of DIC in malaria was 11.6% (95% CI: 8.9–14.3%, I^2^: 93.2%, 38 studies, Figure 2). The meta-regression analysis using publication years, study design, continent, age group, *Plasmodium* spp., method for malaria detection, and clinical status showed that the study design, clinical status, and types of complications were the probable sources of heterogeneity of the proportion estimates between the studies (*p* = 0.03, *p* = 0.03, and *p* < 0.01, respectively, Table S3). 

**Figure 3:**
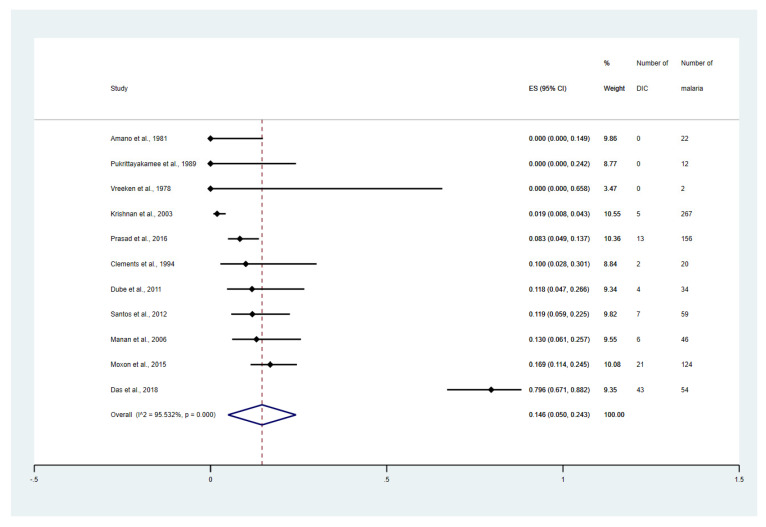
Proportion estimates of DIC among patients with severe malaria. The figure shows the proportion estimates of DIC in patients with severe malaria in an individual study (×100 unit). Abbreviations: DIC, disseminated intravascular coagulation; CI, confidence interval; ES, proportion estimates; I^2^, inconsistency index; *p*, significance value of Chi-square test for heterogeneity; % weight, contribution of individual study to the pooled proportion estimate [27,31,32,33,40,43,47,50,51,52,56].

The meta-analyses stratified by the study design, clinical status, *Plasmodium* species, and types of complications were performed. The meta-analysis demonstrated that the overall proportion estimate of DIC in malaria among the cohort studies, retrospective observational studies, cross-sectional studies, and prospective observational studies were 21.0% (95% CI: 5.9–36.1%, I^2^: 98.20%, 5 studies, Supplementary Figure S1), 10.9% (95% CI: 6.4–15.5%, I^2^: 78.43%, 10 studies, Supplementary Figure S2), 9.6% (95% CI: 4.5–14.7%, I^2^: 89.9%, 12 studies, Supplementary Figure S3), and 6.9% (95% CI: 3.0–10.8%, I^2^: 90.51%, 10 studies, Supplementary Figure S4), respectively. The meta-analysis involving different levels of severity of *P. falciparum* infections was performed. The result showed that the overall proportion estimates of DIC among patients with non-severe falciparum malaria was 0%, (Supplementary Figure S5). Meanwhile, the overall proportion estimates of DIC among patients with severe falciparum malaria was 14.6% (95% CI: 5.0–24.3%, I^2^: 95.5%, 11 studies, Figure 3). The overall proportion estimates of DIC among patients with fatal malaria was 82.2% (95% CI: 56.2–100.0%, I^2^: 87.3, 4 studies, Supplementary Figure S6).

**Figure 4:**
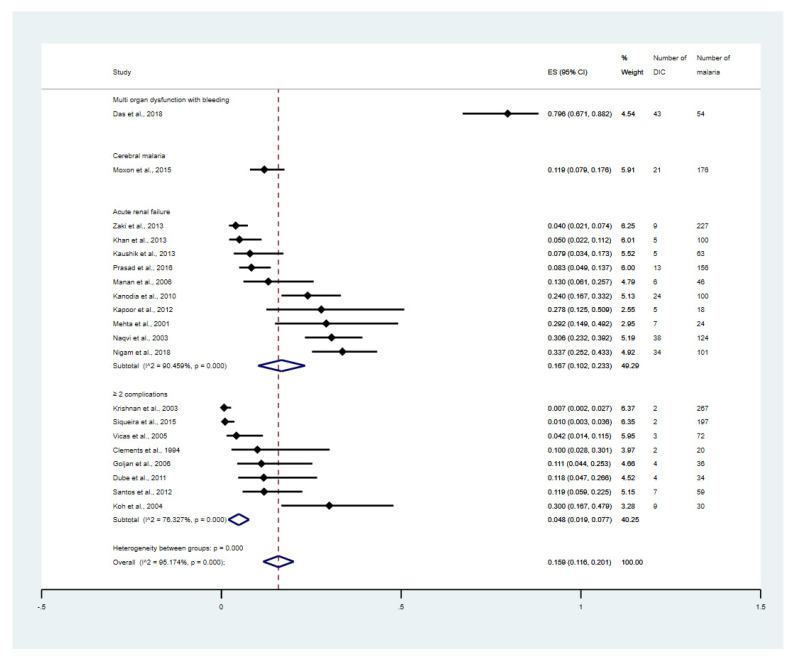
Proportion estimates of DIC among patients with malaria stratified by types of severe complications. The figure shows the proportion estimates of DIC in patients with malaria in an individual study (×100 unit) and the pooled proportion estimates of DIC in patients with malaria in each group, including cerebral malaria, acute renal failure, and more than one complication. Abbreviations: DIC, disseminated intravascular coagulation; CI, confidence interval; ES, proportion estimates; I^2^, inconsistency index; *p*, significance value of Chi-square test for heterogeneity; % weight, contribution of individual study to the pooled proportion estimate; ≥2 complications, study that enrolled patients with severe malaria that having at least two complications [31,32,33,34,36,37,38,39,40,43,47,48,49,50,52,53,55,58,60,61].

The subgroup meta-analysis of severe complications was performed. The results showed that the overall proportion estimates of DIC among the severe malaria patients who had multi-organ dysfunction with bleeding, cerebral malaria, acute renal failure, and ≥2 complications were 79.6% (95% CI: 67.1–88.2%, 1 study), 11.9% (95% CI: 7.9–17.6%, 1 study), 16.7% (95% CI: 10.2–23.3%, 10 studies), and 4.8% (95% CI: 1.9–7.7%, 8 studies, Figure 4), respectively. The meta-analysis involving *P. falciparum* only versus the studies on the patients with *P. vivax* only was performed. The result showed that the overall proportion estimates of DIC among the patients with *P. falciparum* malaria was 16% (95% CI: 9.6–22.5%, 16 studies, Supplementary Figure S7). Meanwhile, the overall proportion estimates of DIC among the patients with *P. vivax* malaria was 3% (95% CI: 0–6.9%, 3 studies, Supplementary Figure S8). 

### 3.5. Publication Bias

**Figure 5:**
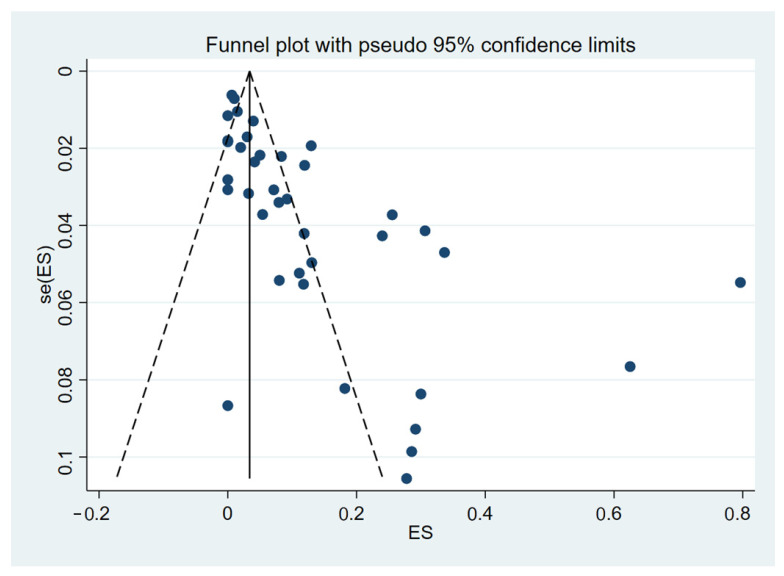
The funnel plot between proportion estimates of DIC in patients with malaria (X-axis) and standard error (se) of the proportion estimates (Y-axis). The plot shows an asymmetry of proportion estimates in the studies included for the meta-analysis as proportion estimates distributed unequally between the middle line (the pooled proportion estimate, black-vertical line). Abbreviations: ES proportion estimate [25,26,27,28,29,30,31,32,33,34,35,36,37,38,39,40,41,42,43,44,45,46,47,48,49,50,51,52,53,54,55,56,57,58,59,60,61,62].

The funnel plot was asymmetrical (Figure 5) and the Egger’s test demonstrated a significance (*p* < 0.01), indicating the existence of a publication bias in the meta-analysis due to a small-study effect or the fact that some studies were missing from the meta-analysis.

## 4. Discussion

Although the epidemiology of DIC in patients with malaria has been reported in the literature, the real evidence of DIC among malaria patients remains unclear. The present study intended to synthesize the evidence of DIC among malaria patients using data from the literature. The results showed that the pooled estimate of the number of patients with malaria who had DIC was 11.6%. The high proportion of DIC among the patients with malaria indicated a likelihood and a probability of bleeding tendency among the individuals who get infected with *Plasmodium* parasites, particularly those patients who developed severe malaria.

DIC usually occurs in severe *Plasmodium* infection with an increased risk of mortality and it can be triggered by several pathological mechanisms, including parasite-derived factors, host immune responses, and endothelial damage that can lead to coagulation activation, coagulation inhibitor defect, and impaired fibrinolysis. Endothelial activation is considered a major pathophysiological feature of malarial infection, which is caused by the interaction of parasite-derived molecules with endothelial receptors, resulting in increased expression of adhesion molecules such as von Willebrand factor (vWF) and vWF propeptides [63]. Hollestelle et al. demonstrated that both vWF and its propeptide levels increased in mild, non-cerebral severe, and cerebral malaria patients [64]. vWF plays a significant role in primary hemostasis by triggering platelet adhesion to the site of damaged endothelial tissue and subsequently inducing platelet aggregation [65]. Increased platelet activation in DIC is mainly caused by the interaction of activated endothelium and the direct action of thrombin on platelets [66]. In malaria infection, anti-platelet antibodies are produced and have been suggested to be partially responsible for platelet activation and leading to thrombocytopenia by platelet destruction through the reticuloendothelial system. Moreover, adenosine diphosphate (ADP), a platelet adhesive cofactor released from the hemolysis of infected red blood cells, induces platelet activation and enhances platelet secretory activity, which results in persistent activation causing platelet dysfunction [8]. In addition, oxidative stress may induce premature platelet destruction through lipid peroxidation in malaria infection, leading to thrombocytopenia [67]. 

The activation of the pro-coagulation stage may occur in mild and severe malaria infections caused by various mechanisms. Phosphatidylserine exposure on the infected red blood cell surface membrane triggers the formation of prothrombinase complex [68]. Pro-inflammatory cytokines, especially tumor necrosis factor-alpha (TNF-α) and interleukin-6 (IL-6), are additional factors in coagulation activation that induce tissue factor expression on monocytes and damaged endothelial cells, primarily through a transcriptional mechanism. In addition, tissue factor is also released from monocytes and platelet-monocyte complexes. The activation of tissue factor enhances the stimulation of the coagulation cascade and thrombin formation [69]. The intrinsic pathway of the coagulation cascade has also been demonstrated to be activated in malaria infection. It may activate the complement system and release bradykinin and polymorphonuclear (PMN)-derived elastase, contributing to the pathogenesis of severe malaria [31]. 

Endogenous anticoagulant proteins, including antithrombin, protein C, and protein S are decreased in malarial infection due to high consumption, leakage, and reduced hepatic production. Under normal physiological conditions, protein C is activated by thrombin and forms a complex with thrombomodulin on the endothelial cell surface leading to proteolytic cleavage of factors Va and VIIIa. However, during malarial infection, protein C is inhibited by inflammatory cytokines [70,71]. Tissue factor pathway inhibitor is another natural anticoagulant mechanism that inhibits tissue factor and factor VIIa complex and induces fibrinolytic action. In addition, the plasma levels of plasminogen activator inhibitor-1 (PAI-1) are elevated in malarial infection and directly correlate with fibrinolysis impairment and increase fibrin formation leading to the deposit of fibrin in the vasculature [72]. 

The high proportion estimate of DIC among patients with malaria found in the present meta-analysis was due to the variability in the study design, clinical severity, and types of severe complications. In the subgroup meta-analysis of different study designs, the proportion estimate of DIC in malaria was highest in the cohort studies (21%). The high proportion estimate (79.6%) was found in the cohort study by Das et al. [32] but the other cohort studies showed the proportion estimate <10% [45,47,50,53]. Das et al. reported a high proportion of DIC among malaria patients with multi-organ dysfunction and overt bleeding, which could explain the high proportion of DIC in this study [32]. In the subgroup meta-analysis of different clinical severities, bleeding was the most severe complication with the highest proportion of DIC. The association between bleeding and DIC was shown in several studies [8,32,33,37,52,73,74]. The bleeding tendency usually occurs late in the disease progression with kidney, lung, and liver complications. In most cases, bleeding is a consequence of DIC with reduced hemostatic regulatory potential associated with thrombocytopenia, consumptive coagulopathy, impaired clotting factor synthesis, and enhanced degradation of the coagulation factors. Consequently, the hemorrhagic tendency may occur, resulting in spontaneous bleeding from various tissues [8,10]. Among complicated malaria cases reported in India, 45% showed signs of overt bleeding from one or multiple sites. In addition, most of them had prolonged prothrombin time (PT), activated partial prothrombin time (aPTT), and increased D-dimer with thrombocytopenia, indicating the manifestation was associated with DIC [32]. 

The meta-analysis involving different levels of severity of *P. falciparum* infections showed that the highest proportion of DIC among the patients was demonstrated in fatal malaria cases (82.2%). The DIC related-death was reported by several studies and linked to *P. falciparum* [28,31,40,44]. In other complications of severe *falciparum* malaria, the proportion of DIC was lower than the fatal malaria cases by 14.6%. Moxon et al. showed that the patients with cerebral malaria had a high proportion of DIC (11.9%) [47]. Cerebral malaria is a fatal neurological complication of malaria characterized by encephalopathy, seizures, retinopathy, and loss of consciousness. Pathogenesis of cerebral malaria is related to the adhesion and accumulation of infected red blood cells in the brain capillaries, the immunological response, endothelial activation, and loss of the blood–brain barrier integrity [75]. The association between cerebral malaria and DIC was explained by several studies [39,44,47,52,63,76,77]. DIC is suggested as the severe complication of cerebral malaria, evident from capillary microthrombi and microhemorrhage in the brain and other organs, such as the lung and kidney, intracerebral hypoxia, and reduced blood flow [44,76]. Laboratory findings demonstrate that plasma D-dimer levels, fibrin degradation products, fibrinogen monomers, and soluble thrombomodulin are elevated in children with cerebral malaria, indicating a hypercoagulable stage [47]. The interactions between parasitized red blood cells and the endothelium induce the cleavage of endothelial protein C receptor (EPCR) and thrombomodulin (TM), which are important components in the protein C anticoagulant pathway. The lower expression of EPCR and TM in cerebral capillaries may lead to functional loss and localized decompensation that results in thrombin production, fibrin deposition, platelet activation, and micro-hemorrhage in cerebral malaria [77]. 

Kidney complication is a common cause of morbidity and mortality in severe malaria infection. The meta-analysis showed that the malarial patients with acute renal failure also had a high proportion of DIC (16.7%). The association between kidney injury and DIC was explained by several studies [34,36,58,78,79,80,81,82]. However, its pathogenic mechanisms are not fully understood. Malaria infection may be associated with renal injury through several mechanisms, including parasitized red blood cell-associated renal microcirculation blockage, immune-mediated glomerular injury, and red blood cell membrane alteration leading to renal hypo-perfusion [82]. Adherence of the parasitized red blood cells to vascular endothelium results in the accumulation of infected red blood cells in the renal capillaries, causing obstruction of blood flow and hemodynamic instability. During endothelial activation, the increased plasma cytokines and inflammatory mediators, such as thromboxane, catecholamines, and endothelin, are additional causes of malaria-associated kidney injury. Immune system activation in malaria infection can activate complements leading to the accumulation of immune complexes causing glomerulonephritis. Red blood cell hemolysis associated with liver dysfunction from hyperbilirubinemia is another possible risk of renal impairment caused by the hepatorenal syndrome. Subsequently, necrosis of the glomeruli and renal tubules can be accelerated in more severe kidney injury resulting in end-stage kidney disease [81]. 

In the different *Plasmodium* species, the meta-analysis showed a higher overall proportion of DIC among the patients with *P. falciparum* malaria than those with *P. vivax* malaria (16% versus 3%). These results indicate that *P. falciparum* infection led to a higher probability of DIC development than *P. vivax*. The DIC due to *P. vivax* was scarcely reported in the literature, and mostly as case reports [83,84]. Although some included studies aimed at investigating DIC in patients with vivax malaria, a small number of patients showed evidence of DIC [37,46,53]. 

The present study had several limitations. First, the limitation on the quality score of the included studies (as the majority of the included studies had moderate-to-low quality scores), may have an impact on the overall proportion estimated in the current study. Second, the heterogeneity of the proportion estimates between the included studies, which remained in the meta-analysis results though the meta-regression and subgroup analyses, indicated that other confounders or some other characteristics of the participants that were unavailable to be retrieved may be the sources of the heterogeneity, but these are still uninvestigated. Third, publication bias due to a small-study effect or because some studies were missing from the meta-analysis was found in the present meta-analysis and may have had an impact on the meta-analysis’s conclusion. More studies are needed to investigate the evidence, probable risk factors, and mechanism of DIC among patients infected with malaria with the different severity levels of malaria or *Plasmodium* species. Finally, the information from this study would provide useful information to guide the management of malaria patients.

## 5. Conclusions

The present study demonstrated that the pooled proportion estimate of DIC among the patients with malaria was 11.6% and varied between 8.9% and 14.3%. The proportion estimates of DIC among the patients with malaria depended on the clinical severity, *Plasmodium* species, and types of severe complications. The information from this study would provide useful information to guide the management of malaria patients. Future studies are needed to investigate the association between *Plasmodium* infection and DIC and to understand the mechanism of malaria-induced DIC.

## Figures and Tables

**Table 1:** Summary feature of studies.

Characteristics	Number (38 Studies)	%
Publication year		
2010–2022	19	50.0
2000–2009	9	23.7
Before 2000	10	26.3
Study designs		
Cross-sectional studies	12	31.6
Prospective observational studies	10	26.3
Retrospective observational studies	10	26.3
Cohort studies	5	13.2
Case-control studies	1	2.63
Continents		
Africa	3	7.89
Malawi	2	
Congo	1	
Asia	27	71.1
India	14	
Pakistan	6	
Thailand	3	
Singapore	2	
Turkey	1	
Malaysia	1	
Europe	4	10.5
Netherlands	1	
Poland	1	
Portugal	1	
Spain	1	
North America (United States)	3	7.89
South America and Asia (Brazil and India)	1	2.63
*Plasmodium* spp.		
*P. falciparum* only	17	44.7
*P. falciparum* and non-*P. falciparum*	18	47.4
*P. vivax* only	3	7.89
Age groups		
Children	4	10.5
Adults	23	60.5
All age groups	8	21.1
Not specified	3	7.89
Clinical status		
Non-severe malaria	3	7.90
Severe and non-severe malaria	13	34.2
Severe malaria	22	57.9
Methods for malaria detection		
Microscopy	25	65.8
Microscopy, RDT	7	18.4
Microscopy, PCR	1	2.63
Microscopy, RDT, QBC	1	2.63
Microscopy, RDT, PCR, ELISA	1	2.63
Not specified	3	7.89

Abbreviations: RDT, Rapid diagnostic test, PCR, Polymerase chain reaction; QBC, quantitative buffy coat assay; ELISA, enzyme-linked immunosorbent assay.

## Data Availability

All data relating to the present study are available in this manuscript and supplementary files.

## Supplementary Materials

The following supporting information can be downloaded at: https://www.mdpi.com/article/10.3390/tropicalmed8060289/s1, Figure S1: Proportion estimates of DIC among patients with malaria in cohort studies. The figure shows the proportion estimates of DIC in patients with malaria in an individual study (×100 unit) and also the pooled proportion estimates of DIC in patients with malaria. Abbreviations: DIC, disseminated intravascular coagulation; CI, confidence interval; ES, proportion estimates; I^2^, inconsistency index; p, significance value of Chi-square test for heterogeneity; % weight, contribution of individual study to the pooled proportion estimate; Figure S2: Proportion estimates of DIC among patients with malaria in retrospective observational studies. The figure shows the proportion estimates of DIC in patients with malaria in an individual study (×100 unit) and also the pooled proportion estimates of DIC in patients with malaria. Abbreviations: DIC, disseminated intravascular coagulation; CI, confidence interval; ES, proportion estimates; I^2^, inconsistency index; p, significance value of Chi-square test for heterogeneity; % weight, contribution of individual study to the pooled proportion estimate; Figure S3: Proportion estimates of DIC among patients with malaria in cross-sectional studies. The figure shows the proportion estimates of DIC in patients with malaria in an individual study (×100 unit) and also the pooled proportion estimates of DIC in patients with malaria. Abbreviations: DIC, disseminated intravascular coagulation; CI, confidence interval; ES, proportion estimates; I^2^, inconsistency index; p, significance value of Chi-square test for heterogeneity; % weight, contribution of individual study to the pooled proportion estimate; Figure S4: Proportion estimates of DIC among patients with malaria in prospective observational studies. The figure shows the proportion estimates of DIC in patients with malaria in an individual study (×100 unit) and also the pooled proportion estimates of DIC in patients with malaria. Abbreviations: DIC, disseminated intravascular coagulation; CI, confidence interval; ES, proportion estimates; I^2^, inconsistency index; p, significance value of Chi-square test for heterogeneity; % weight, contribution of individual study to the pooled proportion estimate; Figure S5: Proportion estimates of DIC among patients with non-severe malaria. The figure shows the proportion estimates of DIC in patients with non-severe malaria in an individual study (×100 unit) and also the pooled proportion estimates of DIC in patients with non-severe malaria. Abbreviations: DIC, disseminated intravascular coagulation; CI, confidence interval; ES, proportion estimates; I^2^, inconsistency index; p, significance value of Chi-square test for heterogeneity; % weight, contribution of individual study to the pooled proportion estimate; Figure S6: Proportion estimates of DIC among patients with fatal malaria. The figure shows the proportion estimates of DIC in patients with fatal malaria in an individual study (×100 unit) and also the pooled proportion estimates of DIC in patients with fatal malaria. Abbreviations: DIC, disseminated intravascular coagulation; CI, confidence interval; ES, proportion estimates; I^2^, inconsistency index; p, significance value of Chi-square test for heterogeneity; % weight, contribution of individual study to the pooled proportion estimate; Figure S7: Proportion estimates of DIC among patients with falciparum malaria. The figure shows the proportion estimates of DIC in patients with falciparum malaria in an individual study (×100 unit) and also the pooled proportion estimates of DIC in patients with falciparum malaria. Abbreviations: DIC, disseminated intravascular coagulation; CI, confidence interval; ES, proportion estimates; I^2^, inconsistency index; p, significance value of Chi-square test for heterogeneity; % weight, contribution of individual study to the pooled proportion estimate; Figure S8: Proportion estimates of DIC among patients with vivax malaria. The figure shows the proportion estimates of DIC in patients with vivax malaria in an individual study (×100 unit) and also the pooled proportion estimates of DIC in patients with vivax malaria. Abbreviations: DIC, disseminated intravascular coagulation; CI, confidence interval; ES, proportion estimates; I^2^, inconsistency index; p, significance value of Chi-square test for heterogeneity; % weight, contribution of individual study to the pooled proportion estimate; Table S1: Search terms; Table S2: Details of the studies_29-4-2023; Table S3: Quality of the included studies_2-4-2023.
