# Supplementary material for: A Systematic Review and Meta-Analysis of the Proportion Estimates of Disseminated Intravascular Coagulation (DIC) in Malaria
Source: Trop Med Infect Dis. 2023 May 23;8(6):289. doi: 10.3390/tropicalmed8060289 (PMC10301305; doi:10.3390/tropicalmed8060289)
Supplement: Supplementary file 1 [file tropicalmed-08-00289-s001.zip › Supplementary Figures.pdf]

A Systematic Review and Meta-Analysis of the Proportion Estimates of Disseminated Intravascular Coagulation (DIC) in Malaria

Thitinat Duangchan 1,2 , Manas Kotepui 1,* , Suriyan Sukati 1,2 , Yanisa Rattanapan 1,2 and KinleyWangdi 3

1 Medical Technology, School of Allied Health Sciences, Walailak University, Tha Sala, Nakhon Si Thammarat 80160, Thailand

2 Hematology and Transfusion Science Research Center, Walailak University, Tha Sala, Nakhon Si Thammarat 80160, Thailand

3 Department of Global Health, National Centre for Epidemiology and Population Health, College of Health and Medicine, Australian National University, Canberra 2601, Australia

* Correspondence: manas.ko@wu.ac.th

Supplementary Figures

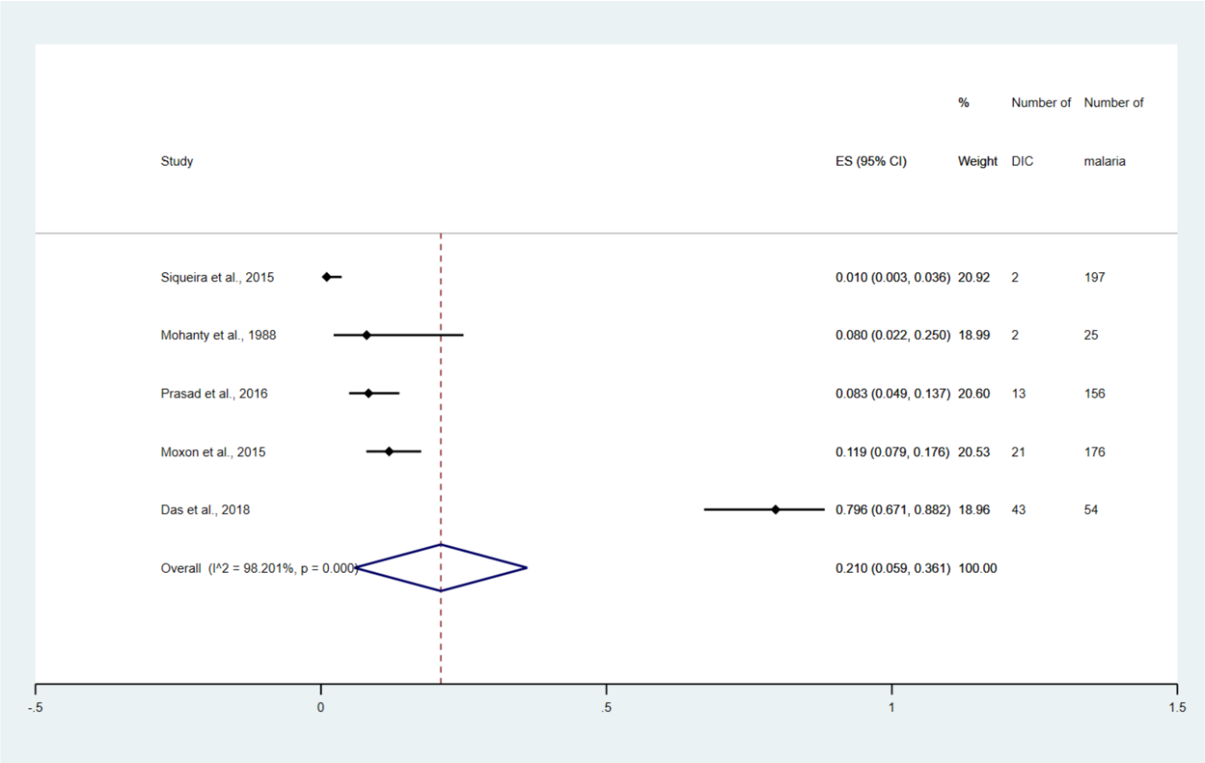

Supplementary Figure S1. Proportion estimates of DIC among patients with malaria in cohort studies. The figure shows the proportion estimates of DIC in patients with malaria in an individual study (x100 unit) and also the pooled proportion estimates of DIC in patients with malaria. Abbreviations: DIC, disseminated intravascular coagulation; CI, confidence interval; ES, proportion estimates; I^2, inconsistency index; p, significance value of Chi-square test for heterogeneity; % weight, contribution of individual study to the pooled proportion estimate.

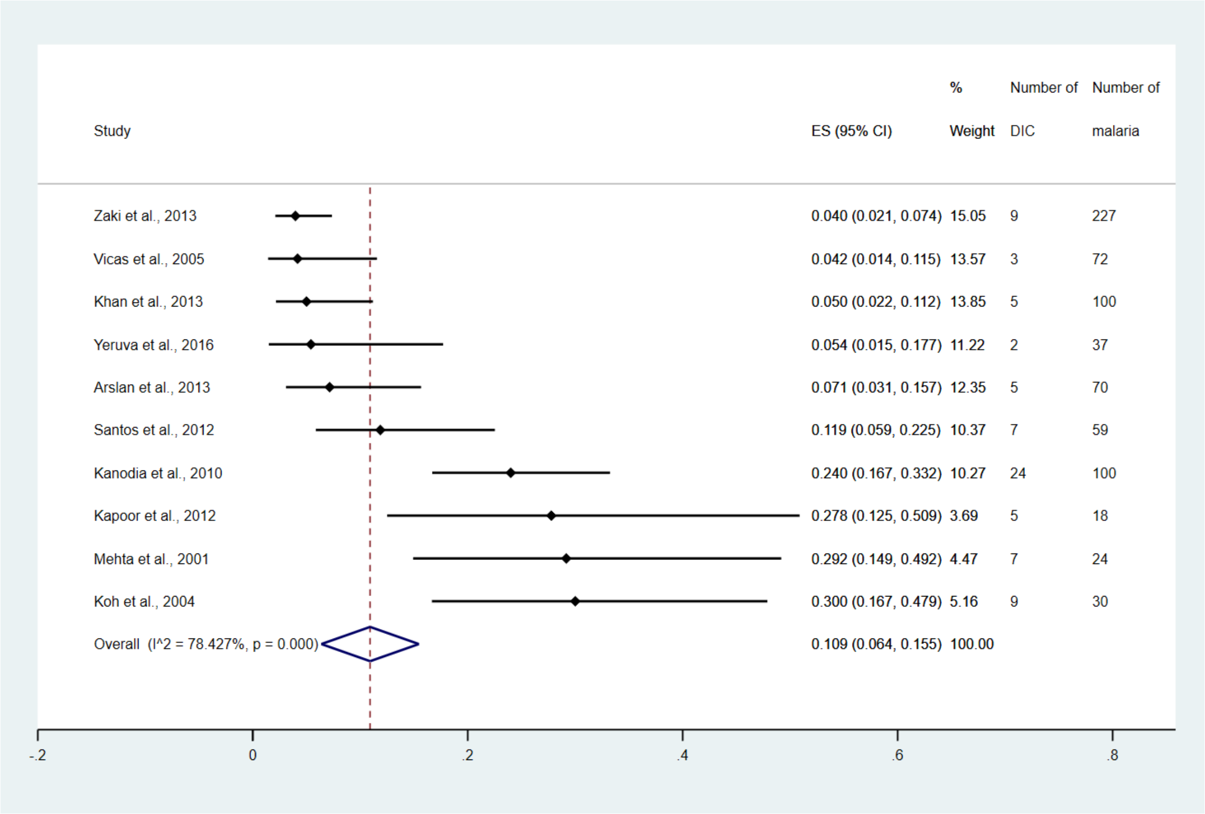

Supplementary Figure S2. Proportion estimates of DIC among patients with malaria in retrospective observational studies. The figure shows the proportion estimates of DIC in patients with malaria in an individual study (x100 unit) and also the pooled proportion estimates of DIC in patients with malaria. Abbreviations: DIC, disseminated intravascular coagulation; CI, confidence interval; ES, proportion estimates; I^2, inconsistency index; p, significance value of Chi-square test for heterogeneity; % weight, contribution of individual study to the pooled proportion estimate.

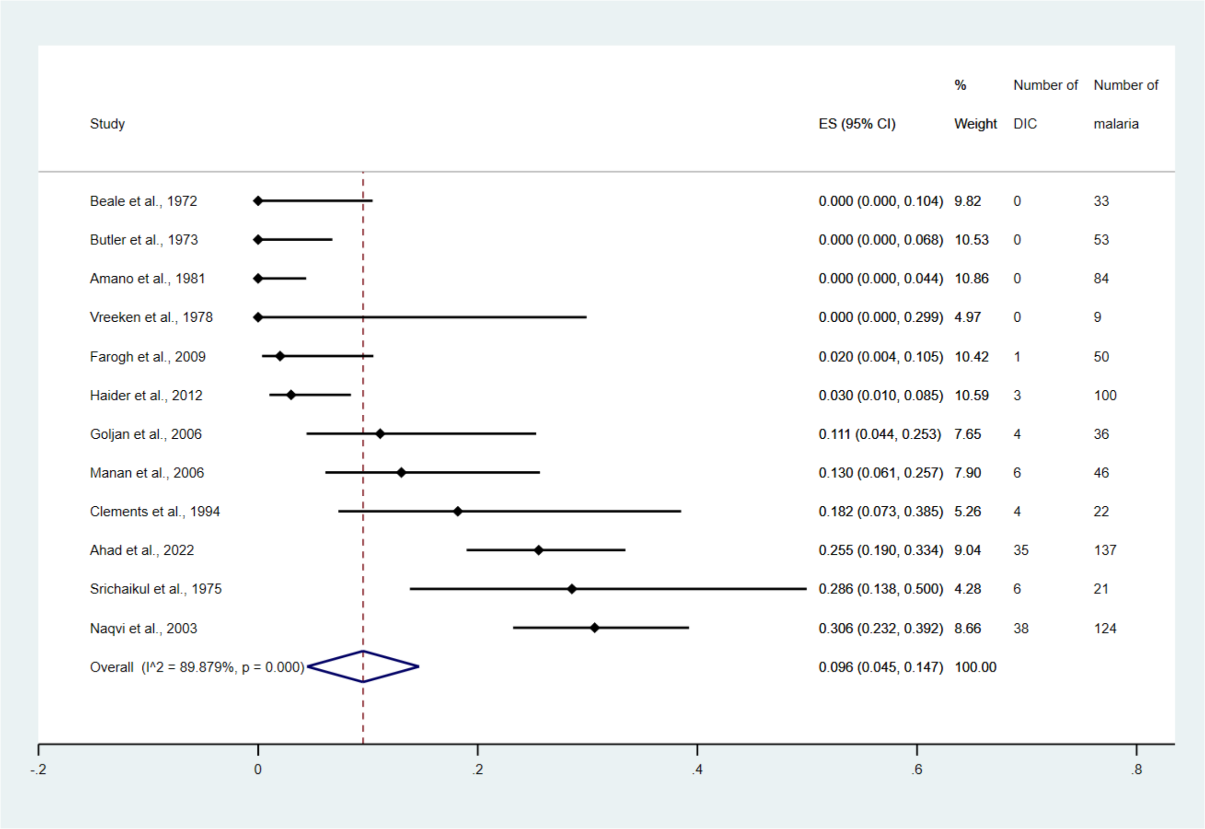

Supplementary Figure S3. Proportion estimates of DIC among patients with malaria in cross-sectional studies. The figure shows the proportion estimates of DIC in patients with malaria in an individual study (x100 unit) and also the pooled proportion estimates of DIC in patients with malaria. Abbreviations: DIC, disseminated intravascular coagulation; CI, confidence interval; ES, proportion estimates; I^2, inconsistency index; p, significance value of Chi-square test for heterogeneity; % weight, contribution of individual study to the pooled proportion estimate.

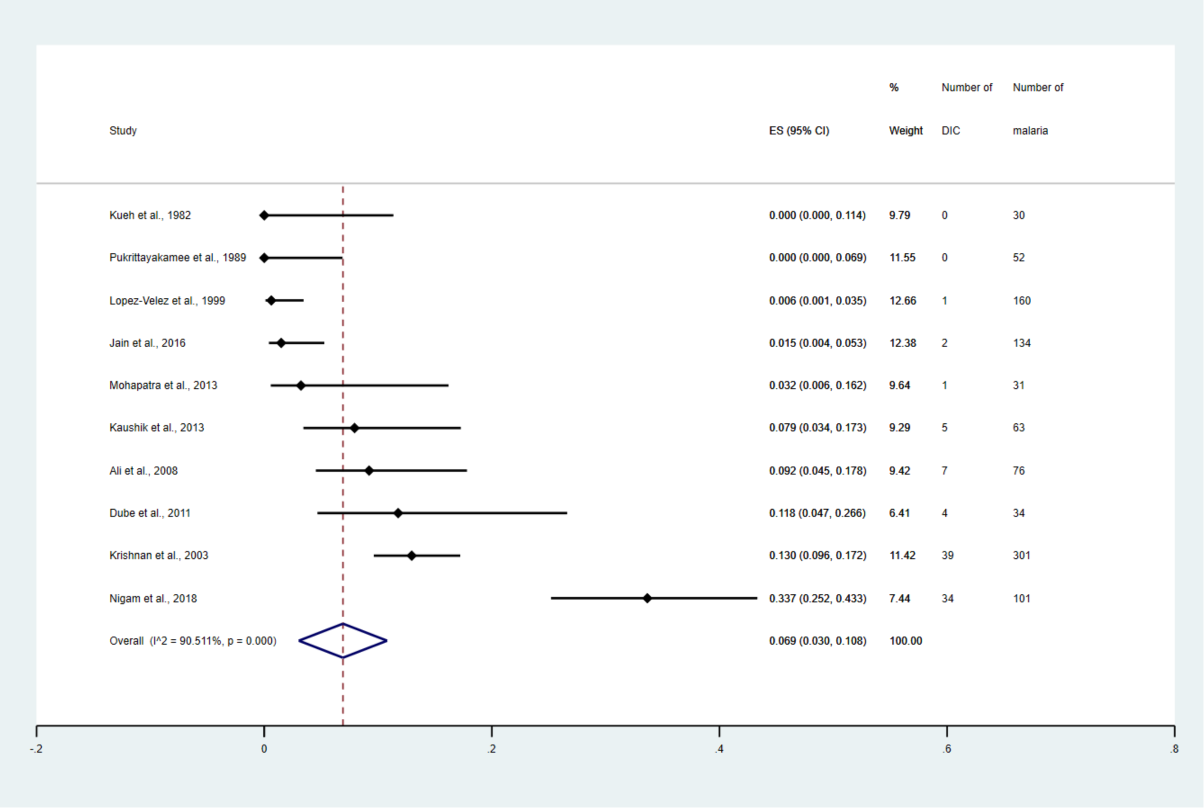

Supplementary Figure S4. Proportion estimates of DIC among patients with malaria in prospective observational studies. The figure shows the proportion estimates of DIC in patients with malaria in an individual study (x100 unit) and also the pooled proportion estimates of DIC in patients with malaria. Abbreviations: DIC, disseminated intravascular coagulation; CI, confidence interval; ES, proportion estimates; I^2, inconsistency index; p, significance value of Chi-square test for heterogeneity; % weight, contribution of individual study to the pooled proportion estimate.

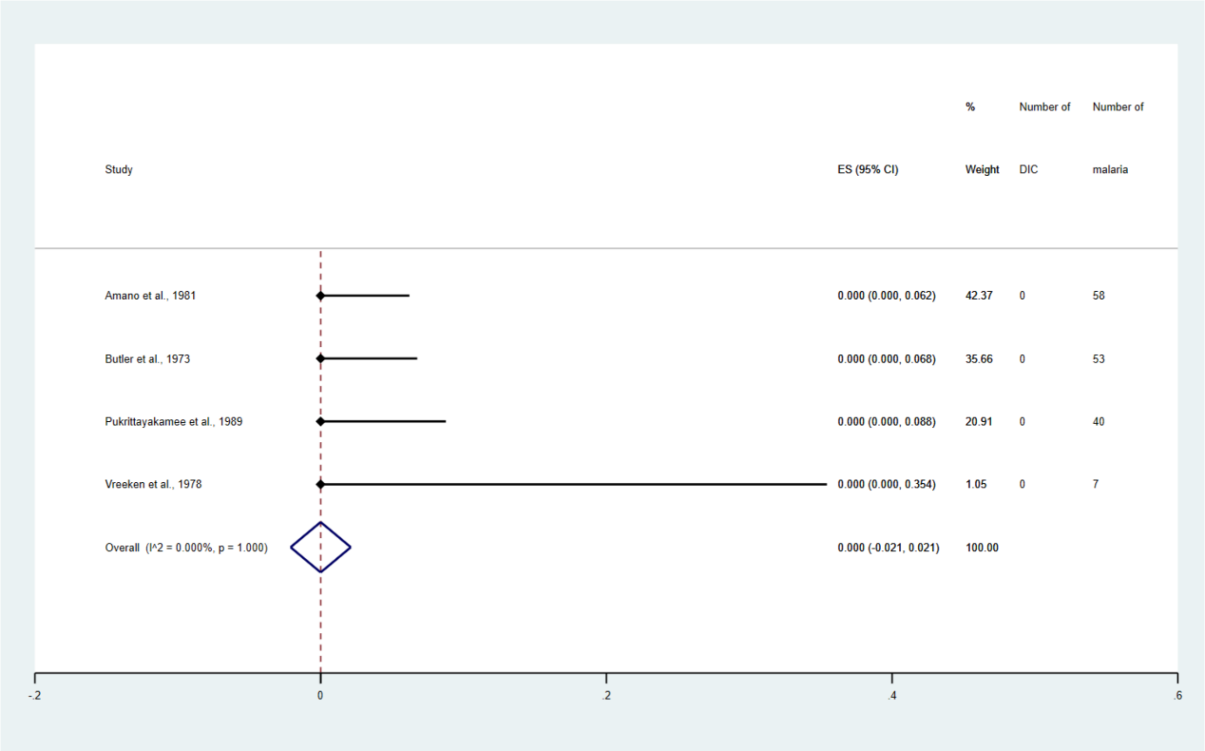

Supplementary Figure S5. Proportion estimates of DIC among patients with non-severe malaria. The figure shows the proportion estimates of DIC in patients with non-severe malaria in an individual study (x100 unit) and also the pooled proportion estimates of DIC in patients with non-severe malaria. Abbreviations: DIC, disseminated intravascular coagulation; CI, confidence interval; ES, proportion estimates; I^2, inconsistency index; p, significance value of Chi-square test for heterogeneity; % weight, contribution of individual study to the pooled proportion estimate.

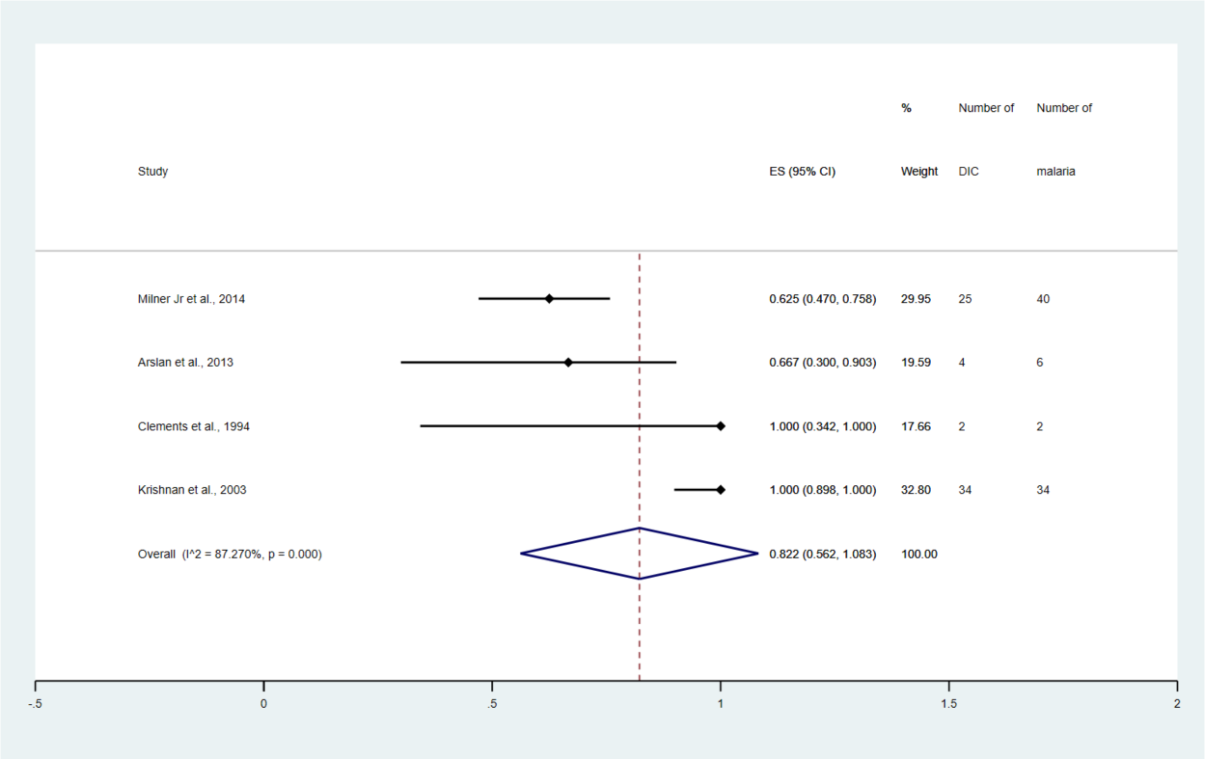

Supplementary Figure S6. Proportion estimates of DIC among patients with fatal malaria. The figure shows the proportion estimates of DIC in patients with fatal malaria in an individual study (x100 unit) and also the pooled proportion estimates of DIC in patients with fatal malaria. Abbreviations: DIC, disseminated intravascular coagulation; CI, confidence interval; ES, proportion estimates; I^2, inconsistency index; p, significance value of Chi-square test for heterogeneity; % weight, contribution of individual study to the pooled proportion estimate.

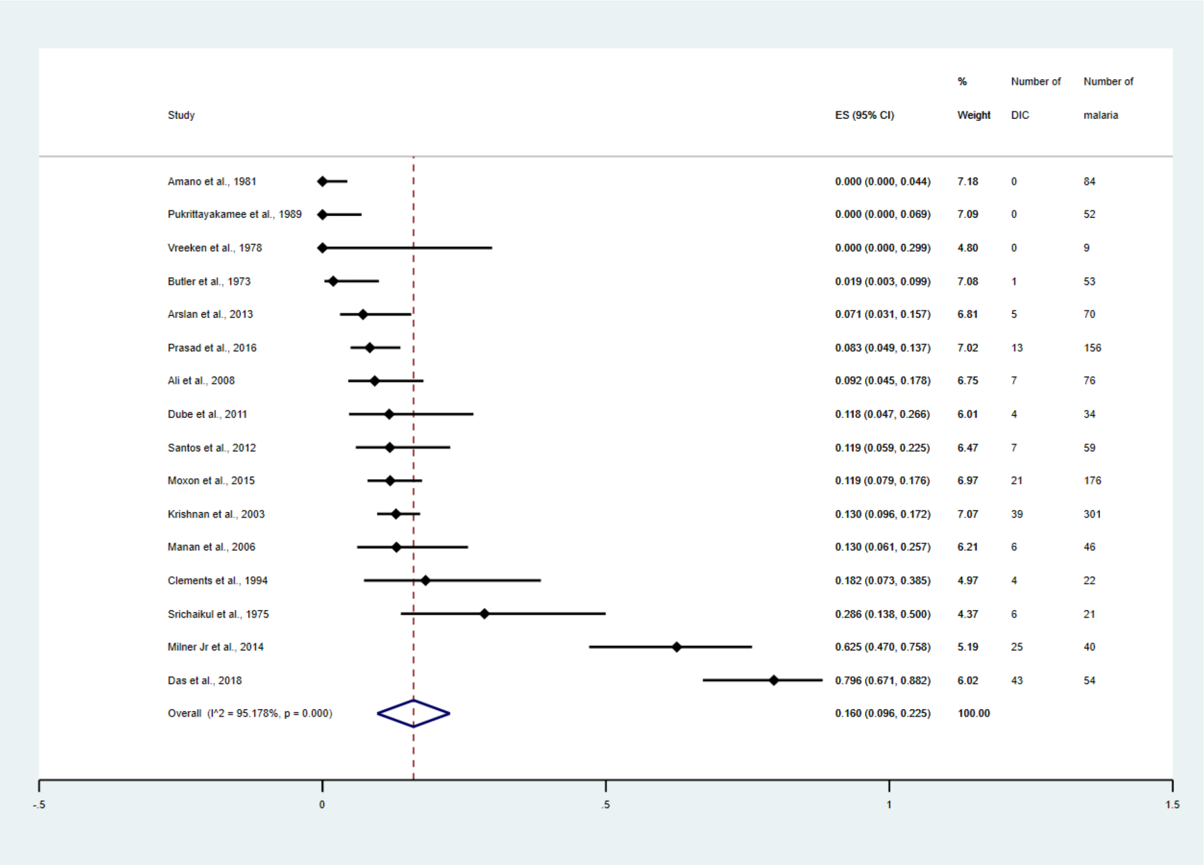

Supplementary Figure S7. Proportion estimates of DIC among patients with falciparum malaria. The figure shows the proportion estimates of DIC in patients with falciparum malaria in an individual study (x100 unit) and also the pooled proportion estimates of DIC in patients with falciparum malaria. Abbreviations: DIC, disseminated intravascular coagulation; CI, confidence interval; ES, proportion estimates; I^2, inconsistency index; p, significance value of Chi-square test for heterogeneity; % weight, contribution of individual study to the pooled proportion estimate.

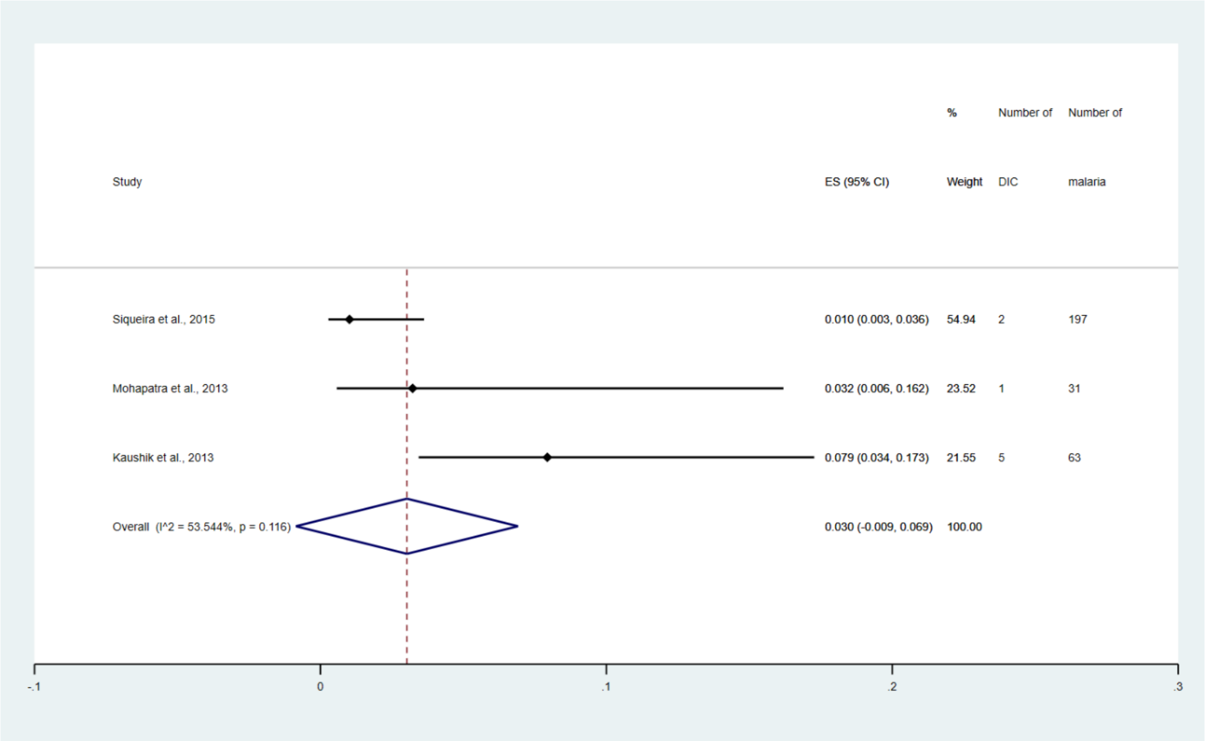

Supplementary Figure S8. Proportion estimates of DIC among patients with vivax malaria. The figure shows the proportion estimates of DIC in patients with vivax malaria in an individual study (x100 unit) and also the pooled proportion estimates of DIC in patients with vivax malaria. Abbreviations: DIC, disseminated intravascular coagulation; CI, confidence interval; ES, proportion estimates; I^2, inconsistency index; p, significance value of Chi-square test for heterogeneity; % weight, contribution of individual study to the pooled proportion estimate.
