# Supplementary material for: A Systematic Review and Meta-Analysis of the Proportion Estimates of Disseminated Intravascular Coagulation (DIC) in Malaria
Source: Trop Med Infect Dis. 2023 May 23;8(6):289. doi: 10.3390/tropicalmed8060289 (PMC10301305; doi:10.3390/tropicalmed8060289)
Supplement: Supplementary file 1 [file tropicalmed-08-00289-s001.zip › Table S1. Search terms.pdf]

A Systematic Review and Meta-Analysis of the Proportion Estimates of Disseminated Intravascular Coagulation (DIC) in Malaria

Thitinat Duangchan [1,2], Manas Kotepui [1,*], Suriyan Sukati [1,2], Yanisa Rattanapan [1,2] and Kinley Wangdi [3]

[1] Medical Technology, School of Allied Health Sciences, Walailak University, Tha Sala, Nakhon Si Thammarat 80160, Thailand; thitinat.du@wu.ac.th (T.D.); suriyan.su@wu.ac.th (S.S.); yanisa.rt@wu.ac.th (Y.R.)
[2] Hematology and Transfusion Science Research Center, Walailak University, Tha Sala, Nakhon Si Thammarat 80160, Thailand
[3] Department of Global Health, National Centre for Epidemiology and Population Health, College of Health and Medicine, Australian National University, Canberra 2601, Australia; kinley.wangdi@anu.edu.au
* Correspondence: manas.ko@wu.ac.th

Table S1. Search strategy

PubMed

7 January 2023

No.	Query	Results
3	#1 AND #2	193
2	(((malaria) OR (malaria[MeSH Terms])) OR (Plasmodium)) OR (Plasmodium[MeSH Terms])	120,400
1	(((Disseminated Intravascular Coagulation) OR (Disseminated Intravascular Coagulation[MeSH Terms])) OR (Consumption Coagulopathy)) OR (Consumption Coagulopathy[MeSH Terms])	17,914

Scopus

7 January 2023

No.	Query	Results
1	(TITLE-ABS-KEY ("disseminated intravascular coagulation" OR "disseminated intravascular" OR "disseminated coagulation" OR "disseminated coagulations" OR "consumption coagulopathy" OR "consumption coagulopathies" OR "intravascular coagulation" OR "intravascular coagulations")) AND (TITLE-ABS-KEY (malaria OR plasmodium OR "remittent fever" OR "marsh fever" OR paludism))	403

	Search results: 403	
2	TITLE-ABS-KEY (malaria OR plasmodium OR "remittent fever" OR "marsh fever" OR paludism)	153,887
1	TITLE-ABS-KEY ("disseminated intravascular coagulation" OR "disseminated intravascular" OR "disseminated coagulation" OR "disseminated coagulations" OR "consumption coagulopathy" OR "consumption coagulopathies" OR "intravascular coagulation" OR "intravascular coagulations")	31,125

MEDLINE

No.	Search terms/Search strategy	Date
1	("Disseminated Intravascular Coagulation" OR "Disseminated Intravascular" OR "Disseminated Coagulation" OR "Disseminated Coagulations" OR "Consumption Coagulopathy" OR "Consumption Coagulopathies" OR "Intravascular Coagulation" OR "Intravascular Coagulations") AND (malaria OR Plasmodium OR "Remittent Fever" OR "Marsh Fever" OR Paludism)<br><br>Search results: 247	7 January 2023

Embase

No.	Search terms/Search strategy	Date
1	('disseminated intravascular coagulation'/exp OR 'disseminated intravascular coagulation' OR 'disseminated intravascular' OR 'disseminated coagulation' OR 'disseminated coagulations' OR 'consumption coagulopathy'/exp OR 'consumption coagulopathy' OR 'consumption coagulopathies' OR 'intravascular coagulation'/exp OR 'intravascular coagulation' OR 'intravascular coagulations') AND ('malaria'/exp OR malaria OR 'plasmodium'/exp OR plasmodium OR 'remittent fever' OR 'marsh fever'/exp OR 'marsh fever' OR 'paludism'/exp OR paludism)<br><br>Search results:418	7 January 2023

Ovid

No.	Search terms/Search strategy	Date
1	("Disseminated Intravascular Coagulation" OR "Disseminated Intravascular" OR "Disseminated Coagulation" OR "Disseminated Coagulations" OR "Consumption Coagulopathy"	7 January 2023

	OR “Consumption Coagulopathies” OR “Intravascular Coagulation” OR “Intravascular Coagulations”) AND (malaria OR Plasmodium OR “Remittent Fever” OR “Marsh Fever” OR Paludism) {Including Limited Related Terms} limit to (ovid full text available and articles with abstracts and original articles)<br><br>Search results: 576	
